# Supplementary material for: Perinatal Environmental Health Education Intervention to Reduce Exposure to Endocrine Disruptors: The PREVED Project
Source: Int J Environ Res Public Health. 2021 Dec 22;19(1):70. doi: 10.3390/ijerph19010070 (PMC8750995; doi:10.3390/ijerph19010070)
Supplement: Supplementary file 1 [file ijerph-19-00070-s001.zip › ijerph-1490198-Supplementary.pdf]

Table S1. Urine biomarkers and exposure, univariate analysis between 2nd (first visit) and 3rd trimester (second visit), subgroup analysis for contextualized and non-contextualized, intent-to-treat analysis, PREVED study.

	Non-Contextualized Group (n = 68)		Contextualized Group (n = 77)		*p*
	**n**	**%**	**n**	**%**	
**Bisphenol A (BPA)**					
Rising indicator	24	*32*	23	*30*	
Same indicator	31	*47*	32	*42*	0.41
Decline indicator	13	*21*	22	*28*	
**BPA Mono-Chlorinated (MCBPA)**					
Rising indicator	19	*28*	15	*19*	
Same indicator	29	*43*	38	*50*	0.47
Decline indicator	20	*29*	24	*31*	
**BPA Di-Chlorinated (DCBPA)**					
Rising indicator	18	*26*	19	*25*	
Same indicator	33	*49*	42	*55*	0.75
Decline indicator	17	*25*	16	*20*	
**BPA Tri-Chlorinated (TCBPA)**					
Rising indicator	11	*16*	11	*14*	
Same indicator	44	*65*	55	*72*	0.66
Decline indicator	13	*19*	11	*14*	
**BPA Tetra-Chlorinated (TTBPA)**					
Rising indicator	15	*22*	7	*9*	
Same indicator	39	*57*	49	*64*	0.09
Decline indicator	14	*21*	21	*27*	
**MethylParaben (MePB)**					
Rising indicator	13	*19*	15	*19*	
Same indicator	34	*50*	37	*48*	0.97
Decline indicator	21	*31*	25	*33*	
**EthylParaben (EtPB)**					
Rising indicator	18	*26*	11	*14*	
Same indicator	27	*40*	32	*42*	0.16
Decline indicator	23	*34*	34	*44*	
**PropylParaben (PrPB)**					
Rising indicator	12	*18*	10	*13*	
Same indicator	46	*68*	53	*69*	0.67
Decline indicator	10	*14*	14	*18*	
**ButylParaben (BuPB)**					
Rising indicator	1	*1*	1	*1*	
Same indicator	65	*96*	74	*96*	0.99
Decline indicator	2	*3*	2	*3*	

Table S2. Colostrum biomarkers and exposure in univariate analysis, intention to treat (ITT), PREVED study.

	Control Group (n BPA = 45) (n Parabens = 39)		Intervention Group (n BPA = 102) (n Parabens = 88)		*p*
	**N**	**%**	**N**	**%**	
**Bisphenol A (BPA)**					
Superior to LoD	34	*76*	79	*77*	0.80

Inferior to LoD	11	*24*	23	*23*	
**BPA Mono-Chlorinated (MCBPA)**					
Superior to LoD	18	*40*	49	*48*	0.37
Inferior to LoD	27	*60*	53	*52*	
**BPA Di-Chlorinated (DCBPA)**					
Superior to LoD	22	*49*	47	*46*	0.75
Inferior to LoD	23	*51*	55	*54*	
**BPA Tri-Chlorinated (TCBPA)**					
Superior to LoD	29	*64*	66	*65*	0.98
Inferior to LoD	16	*36*	36	*35*	
**BPA Tetra-Chlorinated (TTBPA)**					
Superior to LoD	20	*44*	55	*54*	0.29
Inferior to LoD	25	*56*	47	*46*	
**MethylParaben (MePB)**					
Superior to LoD	36	*92*	76	*86*	0.51
Inferior to LoD	3	*8*	12	*14*	
**EthylParaben (EtPB)**					
Superior to LoD	27	*69*	49	*56*	0.15
Inferior to LoD	12	*31*	39	*44*	
**PropylParaben (PrPB)**					
Superior to LoD	13	*33*	26	*30*	0.66
Inferior to LoD	26	*67*	62	*70*	
**ButylParaben (BuPB)**					
Superior to LoD	5	*13*	2	*3*	**0.03**
Inferior to LoD	34	*87*	86	*97*	

LoD : Limit of Detection.

Table S3. Urine biomarkers and exposure , univariate analysis between 2nd (first visit) and 3rd trimester (second visit), intervention group with at least two workshops (Per Protocol analysis) PREVED study.

	**Control Group (n = 93)**		**Intervention Group (n = 132)**		*p*
	**n**	**%**	**n**	**%**	
**Bisphenol A (BPA)**					
Rising indicator	28	*30*	44	*33*	
Same indicator	42	*45*	55	*42*	0.85
Decline indicator	23	*25*	33	*25*	
**BPA Mono-Chlorinated (MCBPA)**					
Rising indicator	17	*18*	32	*24*	
Same indicator	54	*58*	61	*46*	0.21
Decline indicator	22	*24*	39	*30*	
**BPA Di-Chlorinated (DCBPA)**					
Rising indicator	22	*24*	37	*28*	
Same indicator	48	*52*	66	*50*	0.74
Decline indicator	23	*24*	29	*22*	
**BPA Tri-Chlorinated (TCBPA)**					
Rising indicator	15	*16*	20	*15*	
Same indicator	64	*69*	90	*68*	0.94
Decline indicator	14	*15*	22	*17*	
**BPA Tetra-Chlorinated (TTBPA)**					
Rising indicator	17	*18*	19	*14*	
Same indicator	54	*58*	81	*61*	0.73
Decline indicator	22	*24*	32	*25*	

**MethylParaben (MePB)**					
Rising indicator	19	*20*	24	*18*	
Same indicator	56	*60*	65	*49*	0.09
Decline indicator	18	*20*	43	*33*	
**EthylParaben (EtPB)**					
Rising indicator	22	*24*	29	*22*	
Same indicator	42	45	52	39	0.51
Decline indicator	29	31	51	39	
**PropylParaben (PrPB)**					
Rising indicator	11	12	19	14	
Same indicator	73	78	88	67	0.11
Decline indicator	9	10	25	19	
**ButylParaben (BuPB)**					
Rising indicator	3	3	1	1	
Same indicator	89	96	127	96	0.37
Decline indicator	1	1	4	3	
